# Body Mass Shapes Most Life History Traits and a Fast‐Slow Continuum in Amphibians

**DOI:** 10.1002/ece3.70377

**Published:** 2024-10-08

**Authors:** Benjamin Cejp, Eva Maria Griebeler

**Affiliations:** ^1^ Institut für Organismische und Molekulare Evolutionsbiologie Johannes Gutenberg‐Universität Mainz Mainz Germany

**Keywords:** allometric modelling, caecilians, frogs, metabolic theory of ecology, metabolism, salamanders

## Abstract

Amphibians have the least studied life histories among vertebrates, although they have unique and the most diverse life histories within this group. We compiled a new dataset on adult body mass and 16 other life history traits of 2069 amphibian species across three orders (1796 frogs, 236 salamanders, 37 caecilians). These traits characterise fecundity, offspring development from egg deposition to metamorphosis and adult life. We established allometric models on traits for all amphibians and each of the three orders to assess a potential scaling of traits to body mass and then checked whether allometric slopes were consistent with two different metabolic scaling exponents. Further, we examined a possible fast‐slow continuum in all amphibians, as well as in each of the two orders frogs and salamanders by applying principal component analysis (PCA) to five traits. Our allometric models indicated a positive scaling to body mass for 11 traits across all amphibians, 12 in frogs, and 10 in salamanders, and for five out of eight traits analysed in caecilians. Allometric slopes on most traits characterising offspring development were not significant. All slopes did not support a three‐quarter metabolic scaling exponent, whereas slopes on age at maturity and maximum longevity were consistent with an amphibian metabolic scaling exponent of 0.88. As in fishes, reptiles, birds, and mammals, the first axes of our PCAs indicated a body mass‐dependent fast‐slow continuum in amphibians. Amphibian species of slow life histories have larger body masses, later sexual maturities and longer lifespans and lay more and larger eggs than species of fast life histories, a pattern also known from reptiles. The second axes indicated a trade‐off between egg size and number. As this trade‐off was nearly independent of body mass, we hypothesise that amphibians have occupied a broad range of ecological niches without evolutionary changes in body mass.

## Graphical abstract

We examined the effect of body mass on life histories of amphibian species by constructing allometric models based on a large dataset. We found a body mass effect on most traits and found that exponents partly matched those predicted by the metabolic driver hypothesis, but not those predicted by the metabolic theory of ecology. We further identified a body‐mass‐dependent fast‐slow continuum and a nearly mass‐independent trade‐off between egg size and clutch size.

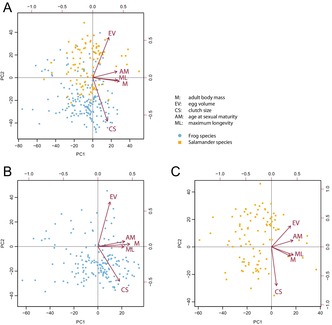

## 1 Introduction

Species show a large variability in their life history traits, for example, they differ in body size, age at onset of reproduction, longevity and offspring number. This variability is reflected in a great diversity in life history strategies, as a life history strategy is the combination of values of life history traits observed in a species (Stearns 1976). These strategies are the result of natural selection, and they ultimately maximise an organism's lifetime reproductive success. Yet, intrinsic constraints and external conditions prevent life history strategies of any species to converge to an evolutionary ideal ‘Darwinian demon’ (Partridge and Harvey 1988). Understanding the association of life history traits seen in species not only adds to an understanding of how their internal constraints have influenced their evolution but also how species have adapted to environmental conditions in the past and how they will adapt in the future.

Empirical studies on vertebrates have shown that many life history traits of species correlate with body size (Peters 1983). Such interspecific correlations between life history traits (TR) and body size (M) of species are quantified by so‐called allometric models. To establish these models, power functions (TR = *a* × M^*b*^) parametrised by an exponent *b* and a normalisation constant *a* are most frequently used (Blueweiss et al. 1978; Peters 1983). After a log–log transformation, a power function yields a linear function (log_10_(TR) = log_10_(*a*) + *b* × log_10_(M)), in which *b* is the slope, and log_10_(*a*) is the intercept of a straight line. Most allometric models are obtained from linear regression analysis of log–log‐transformed data. The exponent of the power function (= slope of the linear function) *b* informs whether TR depends on M (*b* ≠ 0), that is, TR scales to M, or whether TR is independent of M (*b* = 0, TR = *a*), that is, TR does not scale to M. Further, if *b* > 0 TR increases with M, if *b* < 0 TR decreases with M. In the special case *b* = 1, the power function is in fact a linear function with TR/M = *a*. Allometric models with *b* = 1 are termed isometries and those with *b* > 1 hyperallometries. Allometric models can be used as predictive tools.

While this concept of allometric scaling of traits to body size is completely descriptive, its generality across organisms asks for causal explanations. A current hypothesis (hereafter referred to as metabolic driver hypothesis, MDH) on the correlations between body mass and life history traits applies that an organism's metabolic rate determines its rates of biomass production and metabolic turnover times (reviewed in Glazier 2015). Thus, according to the MDH, metabolic rate should determine biomass production‐related life history traits such as clutch mass and individual growth rates, and it should also determine biological time‐related traits such as age at sexual maturity and longevity. The relation between the metabolic rate and the body mass of species is also modelled by a power function and the allometric exponent used is termed the metabolic scaling exponent (Brown et al. 2004; Glazier 2022; Kleiber 1932; Peters 1983). Given that body mass sets metabolic rate, and that in turn metabolic rate determines life history traits, we would expect that exponents of allometric models *b* relating life history traits of species to their body mass can be predicted from the metabolic scaling exponent (*m*) (Brown et al. 2004; Peters 1983). In particular, under the MDH, *b* = *m* is expected for traits related to the individual rate of biomass production (e.g., annual clutch mass), *b* = 1 − *m* for biological time–related traits (e.g., longevity) and *b* = *m* − 1 for biological rate–related traits (e.g., number of clutches per year) (Peters 1983).

While the MDH allows for differences in metabolic scaling exponents among taxa, the metabolic theory of ecology (MTE; Brown et al. 2004) does not. The MTE applies a general metabolic scaling exponent of ¾ in the power function (Kleiber's law; Kleiber 1932), and additionally accounts for temperature by the Arrhenius‐Boltzmann term in order to model the metabolic rate of any organism from its body mass (Brown et al. 2004). The general ¾ power scaling of metabolic rate is supported by the physiological model of West et al. (1997; West, Brown & Enquist's WBE model). For the scaling exponent of life history traits (*b*), the MTE predicts that *b* = ¾ for traits related to the individual rate of biomass production, *b* = ¼ for biological time–related traits and *b* = −¼ for biological rate–related traits (Brown et al. 2004; Peters 1983).

Since it was first proposed, the MTE has been controversial (see, e.g., Glazier 2015; O'Connor et al. 2007; Price et al. 2010). Both the universality of the ¾ scaling of metabolic rate across groups of different taxonomic levels (Glazier 2022; Hatton et al. 2019) and the validity of the WBE model have been doubted (Kozlowski and Konarzewski 2004). For example, White, Phillips, and Seymour (2006) calculated metabolic scaling exponents ranging from 0.64 in birds to 0.88 in amphibians and fishes. While metabolic scaling exponents differing from ¾ do not question that metabolic rate creates the correlations between body mass and life history traits (MDH), they ask at least for alternative explanations than Kleiber's law and the WBE model used by the MTE (Ballesteros et al. 2018; Glazier 2010). In contrast, life history traits that are independent of body mass (*b* = 0) question both the MDH and MTE.

The fast‐slow continuum is another concept on the interrelation of species' life history traits (Jeschke, Gabriel, and Kokko 2018; Jeschke and Kokko 2009). The term fast‐slow continuum was coined by Stearns (1983) and Sæther (1987) from studies on mammals and birds. The empirical association among traits described by this continuum is qualitatively the same as that of the r‐K concept (Pianka 1970), but in contrast to the latter, it is purely descriptive and does not aim to explain it (Jeschke, Gabriel, and Kokko 2018; Jeschke and Kokko 2009). All fast‐slow continua reported for different vertebrate groups (i.e., for fishes, reptiles, birds and mammals) so far are represented by an axis or gradient referring to the pace of life, typically expressed as age at sexual maturity or longevity (Bielby et al. 2007; Clobert, Garland, and Barbault 1998; Jeschke and Kokko 2009; Rochet et al. 2000; Stearns 1983). In studies that had body size in their analyses, larger species always have slower lifestyles than smaller species (Bauwens and Diaz‐Uriarte 1997; Clobert, Garland, and Barbault 1998; Jeschke and Kokko 2009; Stearns 1983). Vertebrate groups differ in how the number of offspring relates to the fast‐slow axis. In some groups, the number of offspring increases from a fast to a slow life history (e.g., in reptiles; Clobert, Garland, and Barbault 1998; Hallmann and Griebeler 2015), while in others it decreases (e.g., in birds and mammals, Jeschke and Kokko 2009). For vertebrates, trade‐offs between fecundity‐related traits were also observed on a second axis that was more or less orthogonal to the fast‐slow axis (Bauwens and Diaz‐Uriarte 1997; Rochet et al. 2000; Stearns 1983). Although some authors have suggested that body size, offspring size, offspring number, age at sexual maturity, and longevity, as well as either generation time or interbirth interval, are the most informative to characterise a fast‐slow continuum for vertebrates (Jeschke, Gabriel, and Kokko 2018; Jeschke and Kokko 2009; Reynolds 2003), the literature is inhomogeneous with respect to the number and sets of life history traits studied (Jeschke and Kokko 2009). This hampers comparative studies on fast‐slow continua across different taxonomic groups.

In vertebrates, there is a strong bias with respect to taxonomic groups in which life history traits, strategies and their evolution have been studied. Life histories of mammals and birds have been investigated most frequently and for longer, and thus, general concepts on life history strategies are often based on findings in these groups (Ricklefs 2000; Sæther 1987; Stearns 1983). Studies on life histories of fishes and reptiles have become more frequent in recent years (Beukhof et al. 2019; Hallmann and Griebeler 2018; Juan‐Jordá et al. 2013), while amphibians are the least studied vertebrates. There are only very few studies analysing amphibian life history traits beyond fecundity‐related traits (Hallmann and Griebeler 2020; Stark and Meiri 2018; Wells 2007). Even in large studies analysing life history strategies across all metazoans, amphibians are often lacking (e.g., Burger, Hou, and Brown 2019; Healy et al. 2019). This is remarkable, as amphibians are a useful vertebrate group to examine patterns in the variability of life histories, because they possess unique, more diverse and more flexible life history traits than those seen in other tetrapods (Babich Morrow, Ernest, and Kerkhoff 2021). For example, some amphibians reach maturity after only a few months, while others take more than 10 years, clutch sizes range from a single egg to tens of thousands of eggs, and maximum life spans range from < 1 year to more than a century (Oliveira et al. 2017; Stark and Meiri 2018). Salamanders in particular show an especially large flexibility in their life histories among and within species and even within populations. Entire life history stages can be shortened, prolonged or skipped altogether, and timing of key events such as metamorphosis and sexual maturation are largely decoupled. This strongly questions the existence of an intrinsically determined life history in amphibian species and suggests a strong influence of environments inhabited by species (Bruce 2003).

The allometric models in Hallmann and Griebeler (2020) indicate that amphibian life histories seem to be much less influenced by body mass than those of other vertebrates. So far, only these authors have systematically established allometries for a large number of amphibian life history traits and body mass. They found that across amphibians, many life history traits are independent of body mass, including such for which the MDH and MTE predict that they depend on mass. However, neither the MDH and MTE nor a fast‐slow continuum has been studied rigorously in amphibians so far.

Indeed, many allometric models established on amphibian life history traits and body mass have shortcomings. For example, the study of Hallmann and Griebeler (2020) was limited by the availability of information on the body mass of species, resulting in the exclusion of many species, for which they had information on other life history traits. Consequently, many of their models are based on small sample sizes, which could have led to inflated confidence intervals of slopes and thus numbers of traits resulting to be independent of body mass. In fact, there are other studies that found a scaling to body mass for traits that Hallmann and Griebeler (2020) reported as independent of body mass (Kaplan and Salthe 1979; Pincheira‐Donoso et al. 2021; Salthe 1969). Moreover, several allometric models on amphibian clutch size and egg size are presented in other studies (Furness, Venditti, and Capellini 2022; Gomez‐Mestre, Pyron, and Wiens 2012; Kupfer et al. 2016; Monroe, South, and Alonzo 2015; Pincheira‐Donoso et al. 2021; Prado and Haddad 2005; Salthe 1969; Wells 2007), but conversely to Hallmann and Griebeler (2020), the majority used species' snout‐vent length (SVL) instead of body mass, because information on SVL is more frequent in the literature (Santini et al. 2018). Yet, the use of SVL hampers any comparison of allometric models within amphibians and to other vertebrate groups due to differences in animal body shapes. Specifically, a comparison of allometric exponents to those predicted by the MDH or MTE is at least questionable when using species' SVL as a predictor.

In our study, we established a new, very large dataset on amphibian life history traits to (1) assess for the largest number of amphibian life history traits studied so far (16 traits) their relation to adult body mass, and we include traits for which a relation has never been studied before. (2) We explore whether established relations between amphibian life history traits and body mass corroborate the MDH or MTE. (3) We check for a potential fast‐slow continuum in amphibian life histories and the contribution of body mass to this continuum. To this end, (1) we built allometric models on each trait and adult body mass. (2) We tested whether allometric slopes on life history traits are consistent with the metabolic scaling exponent of 0.88 that White, Phillips, and Seymour (2006) had found for amphibians (MDH) or the metabolic scaling exponent of ¾ (MTE). (3) We finally ran principal component analyses using five key traits (including adult body mass) that have been suggested to characterise fast‐slow continua in vertebrates (Jeschke, Gabriel, and Kokko 2018; Reynolds 2003). We always analysed the three amphibian orders Anura (frogs), Caudata (salamanders) and Gymnophiona (caecilians) together and separately (Hallmann and Griebeler 2020), if respective sample sizes were sufficient.

## 2 Methods

### 2.1 Life History Data and Phylogenetic Information Used

To create our dataset on amphibian life history traits, we started with that from Hallmann and Griebeler (2020), which is largely based on the AmphiBIO (Oliveira et al. 2017) and Trochet et al. (2014) datasets. We completed and expanded it using other published datasets, field guides and primary literature. Our final dataset covered 2069 amphibian species (1796 frogs, 236 salamanders and 37 caecilians), for which we had information on their adult body mass, adult snout‐vent length (SVL, only for frogs and salamanders) or total length (TL, only for caecilians) and on at least one of 16 other life history traits. The proportions of frog, salamander and caecilian species in our dataset reflect the proportions of these groups among all known amphibian species (7693, 823 and 222 species, respectively; Frost 2024).

In all analyses, we used adult body mass in g (*n* = 2069) as a measure of species' adult body size. For 749 species, masses are direct measurements of animals reported in the literature. For the other 1320 species, we calculated masses from body lengths: For frog and salamander species, we calculated masses from their SVLs using the allometries provided by Santini et al. (2018) (see Babich Morrow, Ernest, and Kerkhoff 2021; Stark and Meiri 2018). Specifically, for frogs, we used their family‐specific equations when such were provided for the species, otherwise we applied their microhabitat‐specific average models. For salamanders, we always used their family‐specific equations relating SVL to body mass. For caecilian species with information on TL, we applied the allometry on TL against body mass provided by Stark and Meiri (2018). We used mean masses and lengths whenever available from our sources (Meiri 2010), averaged data if we had several values on a species and used the midpoint of the minimum and maximum if only ranges were available for a species (Furness, Venditti, and Capellini 2022; Stark and Meiri 2018). Following Allen, Street, and Capellini (2017) and Juan‐Jordá et al. (2013), we preferred female masses and lengths, when sex‐specific data was available. We are aware that many amphibian species show a sexual size dimorphism and that applying size averages across sexes could affect the accuracy of our allometric models and the species' positions in the principal component analyses plots. However, we believe that these inaccuracies are small, as we log10‐transformed all trait data prior to our statistical analyses.

Besides information on adult body size, we compiled data on 16 other life history traits for each species (Table A1 in Appendix A). These are egg diameter in mm (*n* = 1362), clutch size (the number of eggs per clutch, *n* = 1860), annual clutch number (*n* = 35), incubation time in days (time until hatching or birth, *n* = 191), TL of hatchlings in mm (*n* = 105), SVL of hatchlings in mm (*n* = 42), larval period in days (time from hatching to metamorphosis, *n* = 167), TL at metamorphosis in mm (*n* = 204), SVL at metamorphosis in mm (*n* = 64), juvenile mass in g (measured directly after metamorphosis, *n* = 16), size at (sexual) maturity in mm (*n* = 237), age at (sexual) maturity in years (*n* = 418) and maximum longevity in years (*n* = 524). Additionally, we estimated egg volume in cm^3^ (*n* = 1362), as a measure of egg mass; clutch volume in cm^3^ (*n* = 1240), as a measure of clutch mass; and annual clutch volume in cm^3^ (*n* = 33), as a measure of annual clutch mass, from egg diameter (*V* = ^4^/_3_ π r^3^; Bruce 2014; Gould et al. 2022; Kupfer et al. 2016), egg volume times clutch size (Bruce 2014; Kaplan and Salthe 1979) and clutch volume times number of clutches per year, respectively. We averaged trait values, whenever we had values from multiple sources, except for maximum longevity, for which we used the maximum value encountered (Stark and Meiri 2018). Values of TL and SVL at metamorphosis were adopted from literature when it was explicitly stated to be the length at metamorphosis, otherwise values were estimated from the mean length of tadpoles in Gosner‐stages 39–42, in which total length peaks before tail absorption begins. Larval period and TL and SVL at metamorphosis are not applicable to species showing direct development or viviparity, and thus, all analyses on these three traits were exclusively based on species with a larval stage.

Unlike Hallmann and Griebeler (2020), we did not adopt values on the trait ‘reproductive output’ (corresponding to annual clutch number) from the AmphiBIO database (Oliveira et al. 2017), because we consider them unreliable for the following three reasons. (1) The origin of these data is unclear. AmphiBIO provides values on this trait for 4435 of 6776 species. For all these species, Oliveira et al. (2017) give Foden et al. (2013) as the source in which we neither found empirical data, nor did these authors cite other sources on this trait. The authors simply state that ‘species were considered to have low reproductive capacity and hence poor evolvability where they […] have low annual reproductive output (≤ 50 offspring (where known) or they are viviparous)’ (Foden et al. 2013; Supporting Information S1). Note, that Foden et al. (2013) use the term ‘reproductive output’ to describe the annual number of offspring (= clutch size times number of clutches per year), whereas Oliveira et al. (2017) define it as the ‘maximum no. of reproduction events per year’ (= maximum number of clutches per year). (2) The data are likely erroneous. In AmphiBIO, 4372 out of 4435 species (98.6%) have a ‘reproductive output’ of one clutch per year. Despite intense efforts, we could find information on annual clutch number for only 35 species in the literature, and for 29 of these (82.9%) their values did not match 1 as reported in AmphiBIO. In line with that, tropical species, making up the largest proportion of amphibians, generally produce multiple clutches per year (Crump 1974; Duellman and Trueb 1986; Hartmann, Hartmann, and Haddad 2010; Prado and Haddad 2005), whereas many salamanders lay clutches every other year (Duellman and Trueb 1986). (3) It is highly unlikely that values on ‘reproductive output’ given in AmphiBIO are all field data, because AmphiBIO provides values even for species that are only known from few museum specimens (e.g., *Craugastor coffeus*, *Craugastor cruzi*, *Gastrotheca lauzuricae*, *Stefania evansi*, *Nototriton tapanti* or *Noblella peruviana*; IUCN 2023).

All statistical analyses were done in R version 4.2.0 (R Core Team 2023). For our phylogeny‐informed statistics on amphibian life history traits, we applied the large‐scale phylogenetic tree of Jetz and Pyron (2018). We used the Integrated Taxonomic Information System website (itis.gov) to identify synonymous species names and adopted the nomenclature of the tree for our dataset.

### 2.2 Allometric Modelling

For allometric modelling, we first log_10_‐transformed adult body masses and values of all 16 life history traits of species. For each trait, we then fitted a phylogenetic generalised least squares linear regression model using the *gls*‐function of the *nlme*‐package (Pinheiro et al. 2023), following strictly the instructions of Revell and Harmon (2022). We used body mass as a predictor for each of the species' life history traits and a phylogenetic tree pruned to the set of species analysed. We applied Pagel's *λ* (Pagel 1999) to convert our phylogeny into a correlation structure by using the function *corPagel* (*ape*‐package; Paradis, Claude, and Strimmer 2004). We calculated 95% confidence intervals of estimated slopes, intercepts and *λ* values with the function *intervals*. Following Hallmann and Griebeler (2020), we established allometric models using all amphibians from our dataset (hereafter all amphibians together) and using subsamples only consisting of frogs or of salamanders, as these authors found different allometric models on life history traits and adult body mass among these taxonomic groups. Although sample sizes in caecilians were generally small, we further established allometric models for eight traits against body mass, for which we had data on at least four species (egg diameter, egg volume, clutch size, clutch volume, TL of hatchlings, size and age at maturity and maximum longevity). For testing whether statistically non‐zero slopes and intercepts differed between two allometric models, we compared their 95% confidence intervals by applying the method of Smith (1997).

### 2.3 Test of MDH and MTE

For eight of the 16 life history traits, we tested whether slopes of their allometric models were consistent with the MDH or MTE. These eight traits are either rates of biomass production, biological times or biological rates or are products of traits out of these three categories. For the MDH, we applied a metabolic scaling exponent of 0.88 (White, Phillips, and Seymour 2006) and for the MTE ¾ (Brown et al. 2004). In particular, we expected for annual clutch volume (rate of biomass production) that the slope of the allometric model matches the scaling exponent of 0.88 for the MDH and ¾ for the MTE. For incubation time, larval period, age at maturity and maximum longevity (biological times), expected slopes were 0.12 (MDH) and ¼ (MTE), respectively, and for annual clutch number (biological rate) –0.12 (MDH) and –¼ (MTE), respectively. For clutch volume and juvenile mass (rate of biomass production scaling with 0.88 or ¾ multiplied by biological time scaling with 0.12 or ¼; the exponents are summed; Brown et al. 2004; Peters 1983), we expected a slope of 1 for the MDH and MTE. For our statistical test of the MDH and MTE, we checked whether slopes of allometric models on traits differed significantly from zero and whether respective exponents expected for traits under the MDH and MTE were within the slopes' 95% confidence interval.

### 2.4 Fast‐Slow Continuum

**FIGURE A1:**
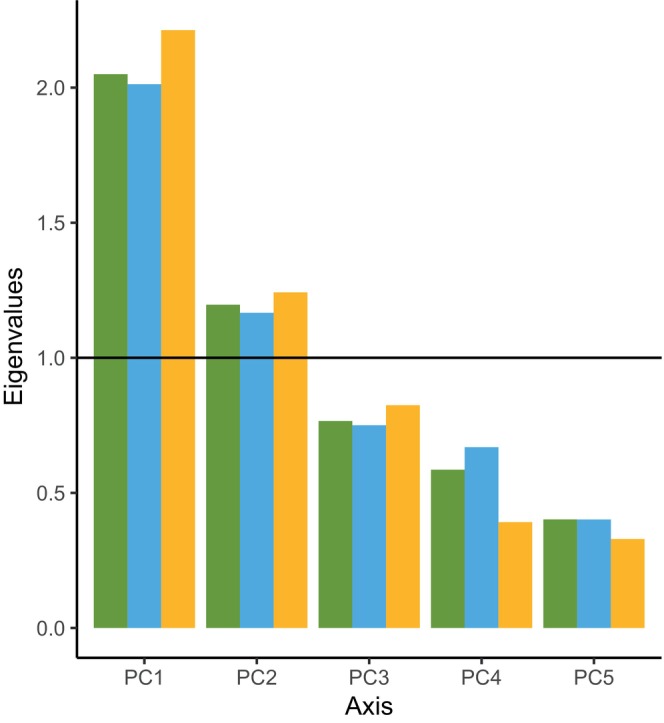
Eigenvalues of all axes shown for each of the three phylogenetic PCAs on the association of five amphibian life history traits. PCAs were conducted with species' adult body mass, egg volume, clutch size, age at sexual maturity and maximum longevity. The horizontal line marks unity and represents the Kaiser–Guttman criterion (Guttman 1954) on the number of axes to be interpreted. Green: All amphibians, blue: Frogs, yellow: Salamanders.

With a subset of 263 species (166 frogs, 97 salamanders) for which we had data on adult body mass, egg volume, clutch size, age at maturity and maximum longevity, we conducted a phylogeny‐informed principal component analysis (PCA) with standardised trait values for all amphibians together, and for frogs and salamanders, separately. These five traits are considered to be the most informative in order to characterise a fast‐slow continuum for a taxonomic group (Jeschke, Gabriel, and Kokko 2018; Reynolds 2003). All PCAs were done with the function *phyl.PCA* from the *phytools*‐package (Revell 2012). We used the phylogenetic tree from Jetz and Pyron (2018) pruned to the analysed species, the method ‘lambda’ (correlation structure derived as for PGLS models) and the mode ‘corr’. The coordinates derived from a phylogeny‐informed PCA still have a phylogenetic signal (Polly et al. 2013). Thus, to test for differences between frogs and salamanders, we ran a phylogenetic MANOVA with the species' PCA coordinates using the function *aov.phylo* (*geiger*‐package; Pennell et al. 2014) and applied a Wilks' *λ* test. We additionally conducted phylogeny‐informed ANOVAs using the function *phylANOVA* (*phytools*‐package) only with the coordinates of the first two PC axes, separately. The Kaiser–Guttman criterion (Guttman 1954) suggested the interpretation of the first two axes (Figure A1 in Appendix A) for all three PCAs.

## 3 Results

### 3.1 Allometric Modelling

We found significant, positive slopes and thus a positive scaling to body mass for 11 out of 16 traits in all amphibians together (Table 1), 12 out of 16 traits in frogs (Table 2), 10 out of 16 traits in salamanders (Table 3), and for five out of eight traits in caecilians (Table 4). Slopes of all other allometric models established in this study were not significant and indicated that the respective trait does not depend on body mass in the respective group of amphibian species.

**TABLE 1:** Allometric models on amphibian life history traits.

Trait	*n*	Slope	95% CI_s_	*p*_s_	Intercept	95% CI_i_	*λ*	References
Egg volume	1362	0.18	0.13, 0.23	**< 0.01**	−1.99	−2.78, −1.19	0.92	This study
Egg mass	14	−0.23	−1.68, 1.21	0.74	−1.39	−1.35, −1.30	1.00	H&G
Egg diameter	1362	0.06	0.04, 0.08	**< 0.01**	0.43	0.17, 0.70	0.92	This study
Egg diameter	327	0.05	0.02, 0.09	**< 0.01**	0.40	0.09, 0.72	0.92	H&G
Clutch size	1860	0.45	0.41, 0.50	**< 0.01**	1.22	0.46, 1.98	0.90	This study
Clutch size	365	0.30	0.21, 0.40	**< 0.01**	1.29	0.34, 2.25	0.91	H&G
Clutch volume	1240	0.71	0.66, 0.77	**< 0.01**	−0.78	−1.20, −0.37	0.49	This study
Annual clutch number	35	0.01	−0.07, 0.08	0.86	0.05	−0.27, 0.38	0.94	This study
Reproductive output	445	< 0.01	−0.01, 0.01	0.92	0.92	0.77, 1.07	0.81	H&G
Annual clutch volume	33	0.12	−0.07, 0.32	0.20	0.63	−0.75, 2.01	1.02	This study
Incubation time	191	−0.03	−0.14, 0.07	0.53	1.56	0.92, 2.19	0.91	This study
Incubation time	57	0.19	−0.01, 0.38	0.06	0.97	0.16, 1.77	0.95	H&G
TL of hatchlings	105	0.10	0.04, 0.16	**< 0.01**	1.19	0.85, 1.52	0.99	This study
SVL of hatchlings	42	0.02	−0.05, 0.10	0.50	0.73	0.36, 1.10	1.04	This study
Duration of the larval period	167	0.07	−0.01, 0.16	0.10	1.99	1.46, 2.51	0.93	This study
Duration of the larval period	39	0.08	−0.17, 0.33	0.53	2.06	1.52, 2.60	0.76	H&G
TL at metamorphosis	204	0.14	0.09, 0.19	**< 0.01**	1.43	1.17, 1.69	0.82	This study
TL at metamorphosis	41	−0.03	−0.20, 0.14	0.72	1.39	1.05, 1.74	0.63	H&G
SVL at metamorphosis	65	0.17	0.11, 0.24	**< 0.01**	1.11	0.84, 1.38	0.84	This study
Juvenile mass	22	0.55	0.28, 0.81	**< 0.01**	−0.70	−1.21, −0.19	0.62	This study
Juvenile mass	16	−0.17	−1.69, 1.34	0.81	0.59	−1.74, 2.91	0.58	H&G
Size at sexual maturity	237	0.21	0.18, 0.24	**< 0.01**	1.68	1.51, 1.85	0.74	This study
Age at sexual maturity	418	0.11	0.07, 0.15	**< 0.01**	0.26	−0.01, 0.53	0.84	This study
Age at sexual maturity	188	0.09	0.05, 0.09	**< 0.01**	0.20	−0.11, 0.20	0.90	H&G
Maximum longevity	524	0.14	0.10, 0.18	**< 0.01**	0.81	0.59, 1.04	0.55	This study
Maximum longevity	189	0.19	0.14, 0.19	**< 0.01**	0.45	0.45, 0.76	0.70	H&G
Maximum longevity	527	0.12	0.06, 0.17	**< 0.01**	0.88	−	0.58	S&M

*Note:* Our models are compared to those in Hallmann and Griebeler (2020) (H&G) and Stark and Meiri (2018) (S&M). Significant *p*‐values are in bold. The phylogenetic signal was significant for all our models.

Abbreviations: *n*, sample size; 95% CI_s_, 95% confidence interval of the slope; *p*_s_, *p*‐value of the slope; 95% CI_i_, 95% confidence interval of the intercept; *λ*, phylogenetic signal assessed from Pagel's *λ* (Pagel 1999).

**TABLE 2:** Allometric models on life history traits of frogs.

Trait	*n*	Slope	95% CI_s_	*p*_s_	Intercept	95% CI_i_	*λ*	References
Egg volume	1174	0.16	0.10, 0.22	**< 0.01**	−2.17	−2.74, −1.61	0.89	This study
Egg mass	9	0.11	−0.63, 0.84	0.73	−1.49	−2.61, −0.36	0.88	H&G
Egg diameter	1174	0.05	0.03, 0.07	**< 0.01**	0.37	0.18, 0.56	0.89	This study
Egg diameter	274	0.05	0.02, 0.09	**< 0.01**	0.35	0.10, 0.60	0.89	H&G
Clutch size	1625	0.49	0.44, 0.54	**< 0.01**	1.76	1.21, 2.31	0.87	This study
Clutch size	304	0.35	0.24, 0.46	**< 0.01**	1.78	1.00, 2.56	0.88	H&G
Clutch volume	1076	0.74	0.68, 0.80	**< 0.01**	−0.54	−0.84, −0.25	0.38	This study
Annual clutch number	14	0.15	0.02, 0.29	**0.03**	0.15	−0.16, 0.47	0.93	This study
Reproductive output	373	< 0.01	0.00, 0.01	0.86	0.03	−0.25, 0.30	1.00	H&G
Annual clutch volume	14	1.26	0.87, 1.64	**< 0.01**	−0.62	−1.54, 0.29	0.95^a^	This study
Incubation time	126	−0.11	−0.24, 0.02	0.09	1.11	0.62, 1.60	0.94	This study
Incubation time	31	0.25	−0.08, 0.57	0.13	0.51	−0.12, 1.14	0.87	H&G
TL of hatchlings	55	0.02	−0.09, 0.13	0.72	0.85	0.55, 1.15	0.97	This study
SVL of hatchlings	28	0.04	−0.09, 0.16	0.54	0.50	0.21, 0.79	1.09	This study
Duration of the larval period	95	0.02	−0.07, 0.11	0.69	1.73	1.40, 2.05	0.92	This study
Duration of the larval period	22	−0.03	−0.18, 0.12	0.72	1.89	1.62, 2.17	0.94	H&G
TL at metamorphosis	115	0.14	0.07, 0.20	**< 0.01**	1.25	1.06, 1.44	0.62	This study
TL at metamorphosis	22	−0.07	−0.32, 0.17	0.55	1.41	1.06, 1.77	0.14	H&G
SVL at metamorphosis	58	0.16	0.10, 0.22	**< 0.01**	0.93	0.81, 1.06	0.52^a^	This study
Juvenile mass	15	0.51	0.18, 0.84	**0.01**	−0.76	−1.28, −0.24	0.62	This study
Juvenile mass	10	−1.31	−2.65, 0.02	< 0.10	2.23	0.03, 4.44	0.91	H&G
Size at sexual maturity	121	0.27	0.24, 0.29	**< 0.01**	1.30	1.26, 1.33	−0.02	This study
Age at sexual maturity	255	0.13	0.08, 0.18	**< 0.01**	0.20	−0.01, 0.41	0.79	This study
Age at sexual maturity	124	0.11	0.05, 0.17	**< 0.01**	0.16	−0.14, 0.46	0.91	H&G
Maximum longevity	365	0.15	0.10, 0.19	**< 0.01**	0.78	0.59, 1.00	0.57	This study
Maximum longevity	125	0.22	0.15, 0.22	**< 0.01**	0.75	0.46, 1.05	0.79	H&G
Maximum longevity	367	0.13	0.07, 0.19	**< 0.01**	0.81	—	0.56	S&M

*Note:* Our models are compared to those in Hallmann and Griebeler (2020) (H&G) and Stark and Meiri (2018) (S&M). Significant *p*‐values are in bold.

Abbreviations: *n*, sample size; 95% CI_s_, 95% confidence interval of the slope; *p*_s_, *p*‐value of the slope; 95% CI_i_, 95% confidence interval of the intercept; *λ*, Phylogenetic signal assessed from Pagel's *λ* (Pagel 1999).

^a^ 95% – confidence interval of λ overlaps with zero.

**TABLE 3:** Allometric models on life history traits of salamanders.

Trait	*n*	Slope	95% CI_s_	*p*_s_	Intercept	95% CI_i_	*λ*	References
Egg volume	173	0.28	0.17, 0.40	**< 0.01**	−1.88	−2.38, −1.37	0.82	This study
Egg diameter	173	0.09	0.06, 0.13	**< 0.01**	0.47	0.30, 0.64	0.82	This study
Egg diameter	53	0.07	0.01, 0.13	**0.04**	0.44	0.01, 0.87	1.00	H&G
Clutch size	203	0.21	0.08, 0.35	**< 0.01**	1.53	0.98, 2.07	0.74	This study
Clutch size	61	0.11	−0.11, 0.33	0.30	1.51	−0.04, 3.05	1.00	H&G
Clutch volume	152	0.50	0.34, 0.66	**< 0.01**	−0.28	−0.78, 0.21	0.56	This study
Annual clutch number	18	−0.04	−0.13, 0.04	0.32	−0.17	−0.35, 0.01	0.47^a^	This study
Reproductive output	72	0.07	0.00, 0.13	0.05	0.44	0.01, 0.88	1.00	H&G
Annual clutch volume	18	−0.01	−0.18, 0.16	0.91	−0.02	−0.91, 0.87	1.03	This study
Incubation time	62	0.08	−0.13, 0.29	0.44	1.62	1.05, 2.19	0.80	This study
Incubation time	26	0.10	−0.12, 0.32	0.35	1.53	0.85, 2.21	1.00	H&G
TL of hatchlings	46	0.14	0.09, 0.19	**< 0.01**	1.10	0.95, 1.25	0.98	This study
SVL of hatchlings	14	0.06	−0.01, 0.14	0.10	1.03	0.93, 1.12	0.87	This study
Duration of the larval period	72	0.14	−0.02, 0.30	0.08	2.27	1.63, 2.92	0.96	This study
Duration of the larval period	17	0.23	−0.30, 0.75	0.37	2.26	1.49, 3.04	0.57	H&G
TL at metamorphosis	89	0.14	0.05, 0.22	**< 0.01**	1.66	1.39, 1.93	0.91	This study
TL at metamorphosis	19	0.02	−0.20, 0.24	0.88	1.37	1.08, 1.65	0.95	H&G
SVL at metamorphosis	7	0.49	0.01, 0.97	**< 0.05**	1.15	0.65, 1.66	0.69^a^	This study
Juvenile mass	6	0.66	−0.62, 1.94	0.23	−0.75	−1.88, 0.38	0.13^a^	This study
Size at sexual maturity	107	0.20	0.14, 0.25	**< 0.01**	1.72	1.56, 1.88	0.58	This study
Age at sexual maturity	156	0.08	0.03, 0.13	**< 0.01**	0.43	0.24, 0.62	0.74	This study
Age at sexual maturity	64	0.04	−0.01, 0.10	0.13	0.42	0.16, 0.68	0.78	H&G
Maximum longevity	153	0.16	0.09, 0.23	**< 0.01**	1.02	0.89, 1.16	0.16^a^	This study
Maximum longevity	64	0.13	0.05, 0.21	**< 0.01**	0.86	0.60, 1.12	0.44	H&G
Maximum longevity	155	0.07	0.05, 0.20	**< 0.01**	0.95	—	0.15	S&M

*Note:* Our models are compared to those in Hallmann and Griebeler (2020) (H&G) and Stark and Meiri (2018) (S&M). Significant *p*‐values are in bold.

Abbreviations: *n*, sample size; 95% CI_s_, 95% confidence interval of the slope; *p*_s_, *p*‐value of the slope; 95% CI_i_, 95% confidence interval of the intercept; *λ*, phylogenetic signal assessed from Pagel's *λ* (Pagel 1999).

^a^ 95% – confidence interval of *λ* overlaps with zero.

**TABLE 4:** Allometric models on life history traits of caecilians.

Trait	*n*	Slope	95% CI_s_	*p*_s_	Intercept	95% CI_i_	*λ*	References
Egg volume	15	0.91	< 0.01, 1.81	**< 0.05**	−2.91	−4.61, −1.21	1.02	This study
Egg diameter	15	0.30	< 0.01, 0.60	**< 0.05**	0.12	−0.44, 0.69	1.02	This study
Clutch size	32	0.22	0.07, 0.36	**< 0.01**	0.80	0.49, 1.11	0.74	This study
Clutch volume	12	0.84	−0.38, 2.06	0.16	−1.53	−3.23, 0.16	−0.30^a^	This study
TL of hatchlings	4	0.31	−0.17, 0.79	0.11	1.54	0.77, 2.32	−1.43^a^	This study
Size at sexual maturity	9	0.12	0.06, 0.18	**< 0.01**	2.24	2.13, 2.34	1.09	This study
Age at sexual maturity	7	0.24	0.09, 0.40	**0.01**	0.03	−0.26, 0.33	1.47	This study
Maximum longevity	6	−0.24	−0.67, 0.19	0.19	1.26	0.33, 2.18	−5.63^a^	This study
Maximum longevity	5	0.27	−1.95, 2.49	0.535	0.56	—	0.00	S&M

*Note:* For maximum longevity, the model from Stark and Meiri (2018) (S&M) is shown. Significant *p*‐values are in bold.

Abbreviations: *n*, sample size; 95% CI_s_, 95% confidence interval of the slope; *p*_s_, *p*‐value of the slope; 95% CI_i_, 95% confidence interval of the intercept; *λ*, phylogenetic signal assessed from Pagel's *λ* (Pagel 1999).

^a^ 95% – confidence interval of *λ* overlaps with zero.

Egg volume, egg diameter, clutch size, size and age at maturity increased significantly with body mass in each of the three orders and in all amphibians together (Tables 1, 2, 3, 4). Clutch volume, TL and SVL at metamorphosis, and maximum longevity increased significantly with body mass in both frogs and salamanders and also in all amphibians together (Tables 1, 2, 3). The SVL of hatchlings, incubation time and larval period did not significantly relate to body mass in frogs and salamanders as well as in all amphibians together (Tables 1, 2, 3). Total length of hatchlings increased significantly with body mass in salamanders, and in all amphibians together, but was independent of body mass in frogs and in caecilians (Tables 1, 2, 3, 4). Annual clutch number and annual clutch volume increased significantly with body mass in frogs and did not relate to body mass in salamanders and all amphibians together (Tables 1, 2, 3). Juvenile mass increased significantly with body mass in frogs and in all amphibians together but did not depend on body mass in salamanders (Tables 1, 2, 3).

For most of our allometric models, we found a significant phylogenetic signal and the majority of *λ* values ranged between 0.8 and 1.0 indicating a Brownian motion model. A phylogenetic signal was not significant for most models that were established on traits with small sample sizes (Tables 1, 2, 3, 4).

### 3.2 Test of the MDH and MTE

For eight traits, we tested whether the slopes of their allometric models are supportive of the MDH or MTE. These traits were clutch volume, annual clutch number, annual clutch volume, incubation time, larval period, juvenile mass, age at maturity and maximum longevity.

When applying a metabolic scaling exponent of 0.88 (White, Phillips, and Seymour 2006), we found seven allometric models for which the exponent expected from the MDH was within the slope's 95% confidence interval (Table 5). In particular, the expected exponent was not rejected for annual clutch volume in frogs, age at maturity in frogs, caecilians and all amphibians together, and for maximum longevity in frogs, salamanders and all amphibians together. In salamanders and all amphibians together, the exponent expected on larval period was also found within the 95% confidence interval, but the lower limit of each interval was slightly negative. For the traits clutch volume, annual clutch number, incubation time, larval period and juvenile mass, we found no statistical support for the MDH for any of the three orders and all amphibians together.

**TABLE 5:** Comparison of slopes of our allometric models on life history traits to exponents expected from the MTE and MDH.

Trait	Group	Slope	95% CI_s_	MTE ex.	White ex.	Support of MTE ex.?	Support of MDH ex.?
Clutch volume^1^	A	**0.71**	**0.66, 0.77**	1	1	NO	NO
Clutch volume^1^	F	**0.74**	**0.68, 0.80**	1	1	NO	NO
Clutch volume^1^	S	**0.50**	**0.34, 0.66**	1	1	NO	NO
Clutch volume^1^	C	0.84	−0.38, 2.06	1	1	NO	NO
Annual clutch number^2^	A	0.01	−0.07, 0.08	−0.25	−0.12	NO	NO
Annual clutch number^2^	F	**0.15**	**0.02, 0.29**	−0.25	−0.12	NO	NO
Annual clutch number^2^	S	−0.04	−0.13, 0.04	−0.25	−0.12	NO	NO
Annual clutch volume^3^	A	0.12	−0.07, 0.32	0.75	0.88	NO	NO
Annual clutch volume^3^	F	**1.26**	**0.87, 1.64**	0.75	0.88	NO	YES
Annual clutch volume^3^	S	−0.01	−0.18, 0.16	0.75	0.88	NO	NO
Incubation time^4^	A	−0.03	−0.14, 0.07	0.25	0.12	NO	NO
Incubation time^4^	F	−0.11	−0.23, 0.02	0.25	0.12	NO	NO
Incubation time^4^	S	0.08	−0.13, 0.32	0.25	0.12	NO	NO
Duration of the larval period^4^	A	0.07	−0.01, 0.16	0.25	0.12	NO	NO
Duration of the larval period^4^	F	0.02	−0.07, 0.11	0.25	0.12	NO	NO
Duration of the larval period^4^	S	0.14	−0.02, 0.30	0.25	0.12	NO	NO
Juvenile mass^1^	A	**0.55**	**0.28, 0.81**	1	1	NO	NO
Juvenile mass^1^	F	**0.51**	**0.18, 0.84**	1	1	NO	NO
Juvenile mass^1^	S	0.66	−0.62, 1.94	1	1	NO	NO
Age at sexual maturity^4^	A	**0.11**	**0.07, 0.15**	0.25	0.12	NO	YES
Age at sexual maturity^4^	F	**0.13**	**0.08, 0.18**	0.25	0.12	NO	YES
Age at sexual maturity^4^	S	**0.08**	**0.03, 0.10**	0.25	0.12	NO	NO
Age at sexual maturity^4^	C	**0.24**	**0.09, 0.40**	0.25	0.12	YES	YES
Maximum longevity^4^	A	**0.14**	**0.10, 0.18**	0.25	0.12	NO	YES
Maximum longevity^4^	F	**0.15**	**0.10, 0.19**	0.25	0.12	NO	YES
Maximum longevity^4^	S	**0.15**	**0.09, 0.23**	0.25	0.12	NO	YES
Maximum longevity^4^	C	−0.24	−0.67, 0.19	0.25	0.12	NO	NO

*Note:* We assumed a metabolic scaling exponent of ¾ for the MTE (MTE ex.; Peters 1983; Brown et al. 2004) and of 0.88 (White, Phillips, and Seymour 2006; MDH ex.) for the MDH. The allometric exponent observed in this study supports the exponent expected for the trait, when it differs significantly from zero (marked in bold) and the expected exponent is within the 95% confidence interval of the slope of the allometric model (95% CI_s_). Trait categories: ^1^produced biomass; ^2^biological rate; ^3^rate of biomass production; ^4^biological time. Taxonomic groups: A = all amphibians together; F = frogs; S = salamanders; C = caecilians.

None of the allometric slopes obtained for the eight traits corroborated the respective exponent expected from the MTE, irrespective of whether any of the three orders or all amphibians together were analysed. The only exception was age at maturity in caecilians for which the 95% confidence interval of the slope of the allometric model included ¼ (Table 5).

Our allometric model on annual clutch number in frogs had a significant positive slope, which contradicts the MDH and MTE, as both expect a negative exponent for this trait (Table 5).

### 3.3 Fast‐Slow Continuum

**FIGURE 1:**
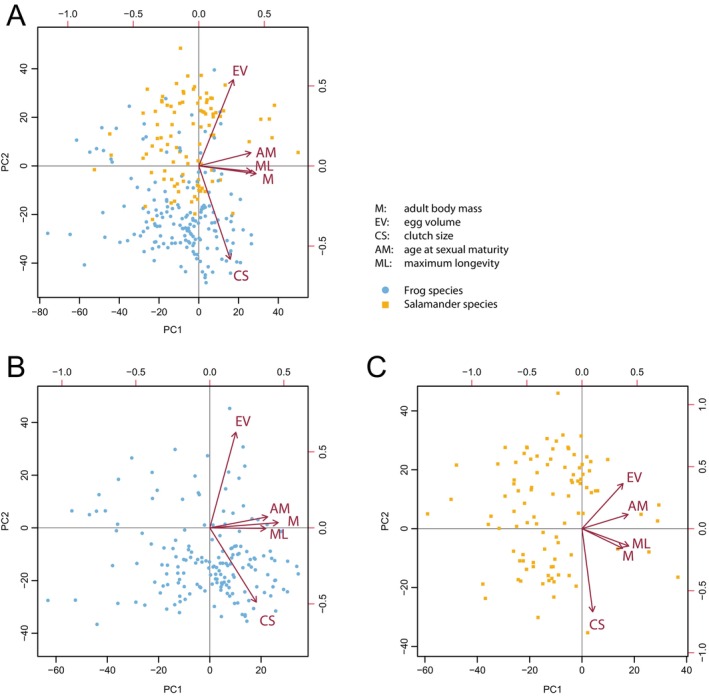
Biplots of phylogenetic PCAs on the association of life history traits in all amphibians (A), frogs (B) and salamanders (C). We used 166 frog and 97 salamander species for which we had values on five traits. Arrows indicate loadings of traits (Table A2 in Appendix A).

The phylogeny‐informed PCAs conducted with frogs and salamanders separately, and with all amphibians together revealed similar results (Figure 1, Table A2 in Appendix A). All five traits of species analysed (adult body mass, egg volume, clutch size, age at maturity and maximum longevity) loaded positively on each of the first PC axes. Adult body mass, maximum longevity and age at maturity contributed the most to all three first axes. Egg volume and clutch size contributed the most to each of the second PC axes. The first two axes of the PCA together explained 64.9% of the total variance in trait associations (Table A2 in Appendix A) when analysing all amphibians together with the first axis contributing 41.0% and the second 23.9% (Table A2 in Appendix A). The phylogenetic signal was large (λ = 0.81). In the order‐specific PCAs, the first two axes together explained 63.6% (PC1: 40.3%, PC2: 23.3%) for frogs and 69.1% (PC1: 44.3%, PC2: 24.8%) for salamanders (Table A2 in Appendix A). The phylogenetic signals were again large, being *λ* = 0.75 in frogs and *λ* = 0.78 in salamanders.

Our three PCAs indicated some differences in the association of the five life history traits between frogs and salamanders. Adult body mass loaded on the first axis the most in frogs, somewhat less in all amphibians together and the least in salamanders (Figure 1, Table A2 in Appendix A). When comparing the two order‐specific PCAs, in the two‐dimensional space, adult body mass, age at maturity and maximum longevity were almost collinear in frogs but were much less collinear in salamanders (Figure 1B,C). In salamanders, egg volume loaded more on the first axis than clutch size, while in frogs it was the other way around (Figure 1B,C). In the PCA on all amphibians together (Figure 1A), salamander species scored higher on the second axis than most frog species. However, associations of life history traits of frogs and salamanders did not differ statistically, neither in the two‐dimensional space (phyl. MANOVA, Wilks' *λ* = 0.55, *p* = 0.43) nor when species' coordinates on PC1 (phyl. ANOVA, *F* = 3.72, *p* = 0.87) and PC2 (phyl. ANOVA, *F* = 203.07, *p* = 0.21) were analysed separately.

## 4 Discussion

Our allometric modelling suggests that more amphibian life history traits correlate to adult body mass than previously thought (Hallmann and Griebeler 2020). Only three out of 16 traits studied did not scale to adult body mass in any of the groups analysed. The slopes of seven allometric models were consistent with the respective exponents expected from the MDH, and only the slope of one model was consistent with that expected from the MTE, favouring a metabolic scaling exponent of 0.88 over ¾ in amphibians. We found a fast‐slow continuum on the association of species' adult body mass, egg volume, clutch size, age at maturity and maximum longevity in frogs, salamanders and all amphibians together. All these interrelations between life history traits found suggest that amphibian life history traits did not evolve independently from each other and that the majority of traits are related to species' adult body mass.

### 4.1 Allometric Modelling

In each of the four groups analysed (frogs, salamanders, caecilians and all amphibians together), the majority of the 16 traits studied scaled positively to adult body mass, that is, the slopes of our allometric models were significant and positive. In particular, seven traits scaled significantly to body mass in all groups in which the trait could be studied. Three traits did not scale to body mass in any of these groups, that is, traits were independent of body mass. The remaining six traits scaled to body mass in some, but not all amphibian groups analysed.

Ten of our 16 traits had been analysed by Hallmann and Griebeler (2020) for frogs and salamanders separately and together. Compared to our results, these authors found that only two traits scaled to mass in all groups. They found that six traits were independent of mass in all groups and that two traits scaled to mass in some, but not all groups. A comparison of allometric models established by Hallmann and Griebeler (2020) and in this study is found in Tables 1, 2, 3, 4.

Compared to Hallmann and Griebeler (2020), our allometric models are based on much larger sample sizes (except for annual clutch number) and are statistically much better constrained. Thus, the non‐significant slopes reported by these authors for nine allometric models are most probably statistical artefacts due to small sample sizes, as we found significant slopes for all these traits using substantially larger samples. These models relate (1) egg mass (frogs), (2) egg mass (all amphibians together), (3) juvenile mass (frogs), (4) juvenile mass (all amphibians together), (5) clutch size (salamanders) (6) age at maturity (salamanders), (7) TL at metamorphosis (frogs), (8) TL at metamorphosis (salamanders) or (9) TL at metamorphosis (all amphibians together) to species' adult body mass. Yet, small sample sizes are relevant for some traits for which we found non‐significant slopes that indicate their independence on body mass. This applies to clutch volume and maximum longevity in caecilians; both scale to body mass in all other taxonomic groups. Furthermore, both egg volume and clutch size, from which clutch volume was calculated, scale to body mass in caecilians. A biased taxonomic composition of species samples might also have resulted in non‐significant allometric slopes found in our study, because such samples likely did not cover the entire variability of a trait in a specific group. In salamanders, our models on annual clutch number and annual clutch volume had non‐significant slopes and our samples on both traits were substantially phylogenetically biased (15 out of 18 species are from one genus). In frogs, we found significant slopes for both traits, even though our sample sizes on frogs were even smaller than those on salamanders, but conversely to salamanders our frog samples were taxonomically well balanced, spanning species from 10 genera and six families. Thus, both the size and the taxonomic composition of a sample are important to produce reliable allometric models.

Our 16 life history traits analysed can be assigned to three categories. These are traits characterising (1) fecundity, (2) development of offspring from egg deposition to metamorphosis and (3) adult life. Each trait category is discussed one after the other in the following three paragraphs.

#### 4.1.1 Traits Characterising Fecundity

The six traits related to fecundity (egg diameter, egg volume, clutch size, clutch volume, annual clutch number and annual clutch volume) were all positively correlated to adult body mass, except for clutch volume in caecilians, and for both annual clutch number and annual clutch volume in salamanders and in all amphibians together. Thus, we confirm previous studies reporting that egg diameter and clutch size increase with body size in all three orders (Gomez‐Mestre, Pyron, and Wiens 2012; Hallmann and Griebeler 2020; Kupfer et al. 2016; Pincheira‐Donoso et al. 2021; Salthe 1969; Wells 2007), but we challenge previous studies finding no relation of body size to egg mass (proportional to egg volume) and egg diameter in frogs, and to clutch size in salamanders (Hallmann and Griebeler 2020; Hartmann, Hartmann, and Haddad 2010; Silva et al. 2020). The positive relations that we found suggest that body size is an important constraint on a species' reproductive capacity.

Clutch volume increased significantly with body mass in frogs, salamanders and in all amphibians together, but not in caecilians. Several studies corroborate this positive scaling of clutch volume to body mass in amphibians. Prado and Haddad (2005) showed for a small sample of frog species that ovary mass, which equates to clutch volume, increases with body size. Salthe (1969) and Kaplan and Salthe (1979) observed for a small sample on salamander species that clutch volume increases with body size. Slopes on clutch volume smaller than 1 found in frogs, salamanders, and all amphibians together in our study confirm that the relative reproductive investment per clutch decreases with increasing body size in all these groups (Kaplan and Salthe 1979; Prado and Haddad 2005; Salthe 1969). The latter observation could reflect that larger species have a longer reproductive life span which in turn allows for more reproductive attempts per lifetime than in smaller species of shorter reproductive life spans. To the best of our knowledge this hypothesis has not been studied for amphibians so far.

Both annual clutch number and annual clutch volume increased with body mass in frogs, but not in salamanders, and in all amphibians together (no data for caecilians). Hallmann and Griebeler (2020) did not find a scaling of annual clutch number to body mass in any of these groups, but their values on annual clutch number were based on the trait ‘reproductive output’ given in the AmphiBIO dataset (Oliveira et al. 2017). We believe that larger species may produce more clutches per year than smaller species, given that the relative investment per clutch decreases with body mass in frogs (see above). For annual clutch volume, our model's hyperallometric slope (> 1) found in frogs suggests that their relative reproductive investment per year increases with body mass across species (interspecific level). For the intraspecific level, such a hyperallometric scaling of fecundity is known for frogs and is common in fishes (Barneche et al. 2018; Gibbons and McCarthy 1986). In fishes, it has been explained by larger animals having advantages in resource acquisition compared to smaller ones (Potter and Felmy 2022), which also may work at the interspecific level. However, the lower limit of the 95% confidence interval of the slope in our allometric model is smaller than 1, which would question a hyperallometric scaling of annual clutch volume in frogs.

#### 4.1.2 Traits Characterising Development of Offspring From Egg Deposition to Metamorphosis

Seven of our 16 traits characterise development of offspring from egg deposition to metamorphosis. These are incubation time, TL and SVL of hatchlings, larval period, TL and SVL at metamorphosis and juvenile mass. For all these traits, we had no data on caecilian species, expect for TL of hatchlings. Five traits showed qualitatively the same relation to body mass in frogs, salamanders and all amphibians together: incubation time, SVL of hatchlings and larval period did not scale to body mass in any of these three groups, whereas TL and SVL at metamorphosis scaled significantly to body mass in each of these groups. The TL of hatchlings, however, increased with body mass in salamanders and in all amphibians together but was independent of body mass in frogs and caecilians. Juvenile mass increased with body mass in frogs and in all amphibians together but did not depend on body mass in salamanders. Our results on incubation time and larval period corroborate the finding of Hallmann and Griebeler (2020) that both do not scale to mass in all groups. That TL at metamorphosis scales to body mass in all groups, however, contradicts Hallmann and Griebeler (2020), who found that this trait is independent of mass in all these groups. Using body length as a predictor of body mass, Salthe (1969) also observed that size at metamorphosis increases with adult size in salamanders, and Werner (1986) reported an increase in size at metamorphosis for ranid and hylid frogs, but not for bufonids. Our finding that incubation time and larval period do not scale to body mass could reflect that these two traits are strongly influenced by both environmental conditions and the reproductive mode of the species. Evidence for our interpretation includes the effect of egg deposition site and ambient temperature on incubation time (Crump 1974; Duellman and Trueb 1986). Further, incubation time is longer in direct developing than in biphasic species, as direct developers stay inside the egg until metamorphosis (Callery, Fang, and Elinson 2001). The larval period of many species depends on external conditions, such as temperature, desiccation risk or food availability in the larval habitat (Crump 2015; Dahl et al. 2012). Many salamanders can stay their entire life in the water and mature as a larva, and this is triggered by external conditions (facultative paedomorphosis; Bruce 2003). That larval period does not scale to adult body mass in frogs, salamanders and all amphibians together seems to contradict our allometric models obtained on other traits. Larval period corresponds to the time needed to achieve size at metamorphosis, and we found that TL and SVL at metamorphosis scale to adult body mass in frogs, salamanders and all amphibians together. Juvenile mass should also relate to larval period as it reflects mass gain during this period, and we found that it increased with adult body mass in frogs and in all amphibians together. That all these size‐related traits scale to adult body mass, but larval period does not, could indicate differences in ontogenetic growth rates of species. For example, a large species having a large size at metamorphosis must not necessarily have a longer larval period than a small species having a small size at metamorphosis, if the growth rate of the large species is higher than the rate of the small one. Fast growth may be advantageous in habitats of high predation risk as large animals face a smaller risk than small animals (Owen‐Smith and Mills 2008; Sinclair, Mduma, and Brashares 2003).

#### 4.1.3 Traits Characterising Adult Life

All three traits characterising adult life (size and age at maturity, and maximum longevity) increased significantly with adult body mass in frogs, salamanders, caecilians and all amphibians together, except for maximum longevity in caecilians. That size and age at maturity increase with body size is reasonable, as it takes longer to achieve larger body sizes and thus sizes at maturity than smaller sizes. For frogs, and all amphibians together, a positive scaling of age at maturity to body mass has been shown before, whereas in salamanders age at maturity was found to be independent of mass (Hallmann and Griebeler 2020). In frogs, salamanders and all amphibians together, an increase in maximum longevity with increasing body mass has also been shown in previous studies (Hallmann and Griebeler 2020; Stark and Meiri 2018).

There are several factors linking adult body mass to longevity. Generally, the attainment of a small adult body mass requires a shorter period of growth than a large mass (Stearns 1992). Larger animals are less prone to predation than smaller animals (Owen‐Smith and Mills 2008; Sinclair, Mduma, and Brashares 2003), resulting in lower extrinsic mortalities and higher longevities in larger than smaller species. This advantage relieves the need for early reproduction in large animals, favouring to slow down their life history intrinsically (Stark, Pincheira‐Donoso, and Meiri 2020). Our allometric models do not statistically support the notion that salamanders are generally longer‐lived than similar‐sized frogs (Duellman and Trueb 1986; Smirina 1994). The slopes and intercepts that we derived for both orders did not differ statistically.

### 4.2 Test of the MDH and MTE

Our test of the MDH and MTE was conducted with clutch volume, annual clutch number, annual clutch volume, incubation time, larval period, juvenile mass, age at maturity and maximum longevity. For these eight traits, we assessed whether slopes of the allometric models established for frogs, salamanders, caecilians and all amphibians together conformed to exponents expected from the MDH and MTE, respectively. While significant differences between allometric slopes and expected exponents question the MDH and MTE, respectively, slopes matching expected exponents do not necessarily prove that metabolic rate drives allometric relations. Our allometric models only demonstrate correlations between life history traits and adult body mass. A similarity of allometric slopes and expected exponents is necessary but not sufficient for causality.

Our finding that all allometric slopes differ statistically from the respective exponents of the MTE (except for age at maturity in caecilians) strongly questions that the metabolic scaling exponent ¾ (Kleiber's law) explains the exponents inferred for the eight traits in each amphibian group.

When applying a metabolic scaling exponent of 0.88 found across amphibians (White, Phillips, and Seymour 2006) to calculate expected exponents, seven significant allometric slopes provided evidence for the MDH. In particular, slopes were consistent with the MDH for annual clutch volume in frogs, age at maturity in frogs, caecilians and all amphibians together, and for maximum longevity in frogs, salamanders and all amphibians together. In salamanders and all amphibians together, the exponent expected for larval period was also found within the respective 95% confidence intervals of the slope, but both slopes were just non‐significant. Whether this supports the MDH or not is unclear. As already discussed above, environmental conditions have a strong influence on the length of larval period in amphibian species (Bruce 2003; Crump 2015; Dahl et al. 2012). A significant influence of body mass on larval period, as predicted by the MDH, might be detectable when controlling for such external factors in allometric modelling.

Interestingly, the majority of allometric slopes supporting the MDH were found for the two biological time–related traits age at maturity and maximum longevity. This could indicate that both traits are substantially affected by metabolic rate in amphibians and thus by an intrinsic process, whereas the two other biological time–related traits studied by us, incubation time and larval period, seem to be more strongly extrinsically affected, that is, by environmental conditions (Crump 2015; Dahl et al. 2012; Duellman and Trueb 1986). We argue that annual clutch volume in frogs, which is expected to be proportional to adult body mass, does not provide support for the MDH. Our sample size on annual clutch volume of frogs comprises only 14 species. Therefore, the 95% confidence interval of the slope of the allometric model was very large (0.87–1.64) and the expected exponent (1) was likely found within this interval. That annual clutch number increases with body mass in frogs, clearly questions the MDH and MTE, as both expect a negative scaling exponent for this trait.

In summary, the slopes derived from our allometric modelling rejected the MTE for amphibians, whereas slopes on age at maturity and maximum longevity supported the MDH in all amphibians together and at least in two orders. However, our test of the MDH was based on a metabolic scaling exponent of 0.88 (White, Phillips, and Seymour 2006) inferred across amphibians. This exponent was derived from ordinary least squares linear regression analysis on metabolic rates and body masses of 158 amphibian species, whereas slopes of all our allometric models are phylogenetically corrected and species samples on many traits, including some on traits for which we found no support for the MDH, were larger. We anticipate that larger samples on metabolic rates of species taken from a broad taxonomic range and analysed by phylogeny‐informed statistics are needed to establish a statistically well‐constrained metabolic scaling exponent for amphibians. This can enable a more rigorous study on whether metabolic rate could drive the variability seen in amphibian life history traits.

### 4.3 Fast‐Slow Continuum

We established fast‐slow continua using the five traits adult body mass, egg volume, clutch size, age at maturity and maximum longevity. In frogs, salamanders and all amphibians together, all five traits were related to the first axis of the respective PCA. This suggests a mass‐dependent fast‐slow continuum for all three groups. Large amphibians have slow life histories, that is, late maturities and long lifespans, whereas small species have fast life histories, that is, early maturities and short lifespans. Amphibians with slower life histories also lay more and larger eggs than amphibians with faster life histories. In all three groups, the fast‐slow continuum is qualitatively consistent with the positive slopes of the respective allometric models. The values of these slopes, however, suggest that egg volume and clutch size are more closely associated with adult body mass than longevity and age at maturity, whereas our PCAs show that the two latter traits are much more closely related to body mass than the two former. These quantitative differences demonstrate that multivariate and not bivariate (allometric) analyses on the association of life history traits should be used to establish potential fast‐slow continua.

The strong intercorrelation seen in the amphibian traits body mass, egg volume, clutch size, age at maturity and maximum longevity as documented by the fast‐slow axis suggests that evolutionary changes due to selection on one or more of these traits go along with predictable changes in all others, changing the entire life history strategy of a species along the fast‐slow axis. For example, an increased body mass selectively favoured in a species simultaneously increases its maximum longevity, egg volume, clutch size and age at maturity.

Further, the fast‐slow continuum indicates that only certain combinations of trait values (i.e., specific life history strategies) have evolved in amphibians. For example, amphibian species of an early maturity and a high fecundity are found at opposite ends of the fast‐slow continuum and no species in our dataset (maybe only very few amphibian species in general) shows both an early maturity and a high fecundity. The absence of this combination of trait values might contribute to that a large proportion of amphibian species is threatened by anthropogenic stressors such as climate change. Species of an early maturity and a high fecundity are able to recover faster from substantial population declines than species of a late maturity and a low fecundity (Reynolds 2003).

In all PCAs, only the traits egg volume and clutch size load substantially on the second axis, which is nearly independent of body mass, age at maturity and maximum longevity. All second axes indicate a trade‐off between egg volume and clutch size as both traits have opposite loadings. This trade‐off likely captures the different reproductive strategies seen in amphibian species (Furness, Venditti, and Capellini 2022; Gould et al. 2022). Different strategies might have enabled species to adapt to environments to some extent even without changing their adult body mass, age at maturity and maximum longevity. This could explain why amphibians comprise a large variability in their reproductive modes and that specific modes have evolved multiple times in their evolutionary history.

Although all our statistical tests on differences in the association of the five life history traits between frogs and salamanders were not significant, our results indicate some differences between both orders. In salamanders, a slow life history is more closely linked to offspring quality (egg volume) than to offspring quantity (clutch size), while the opposite is true for frogs. Our PCA plots further indicate differences in the trade‐off between egg volume and clutch size for frogs and salamanders. This trade‐off arises only when the maximum limit on clutch volume is reached (Dziminski and Alford 2005). However, our PCAs show that in salamanders, the negative relation between egg volume and clutch size is not as strong as in frogs, suggesting that the trade‐off is less pronounced in salamanders than in frogs. This could indicate that salamanders do not maximise the volume of single clutches as they have longer reproductive life spans than frogs, which allows salamanders more breeding attempts within their life than frogs.

To the best of our knowledge, our study is the first showing a fast‐slow continuum for amphibian species. We found a body size‐dependent continuum for all traits, with adult body mass, age at maturity and maximum longevity loading positively and the strongest on a first (fast‐slow) axis. This provides evidence that these three traits represent the most important axis of life history variation across all vertebrate classes (amphibians: this study; reptiles: Clobert, Garland, and Barbault 1998; Hallmann and Griebeler 2015; fishes, birds, and mammals: Jeschke and Kokko 2009) and suggests that there must be a general causal link between adult body mass, age at maturity and maximum longevity. Several authors have suggested that metabolic rate drives the association of these traits in vertebrates as it sets the pace of life (Healy et al. 2019; but see de Magalhães, Costa, and Church 2007; Glazier 2015). That most of our allometric exponents on maximum longevity and age at maturity conform to the MDH provides evidence for this hypothesis.

Vertebrate classes differ mainly in the relation of offspring number (corresponding to clutch size) to the fast‐slow axis. In amphibians and reptiles, clutch size increases from the fast to the slow end of the continuum (this study; Clobert, Garland, and Barbault 1998; Hallmann and Griebeler 2015), while the opposite has been found in mammals, birds and fishes (Jeschke and Kokko 2009; but see Juan‐Jordá et al. 2013 for fishes). Across vertebrates, there are differences whether the trade‐off between egg (offspring) size and clutch (litter) size is nearly mass‐independent, as we found for amphibians, or not (Duarte and Alcaraz 1989; Jeschke and Kokko 2009). This demonstrates that the MDH does not apply to egg (offspring) size and number across all vertebrate classes, because metabolic rate would only determine the total clutch (litter) mass, but not whether this mass consists of few large or many small eggs (offspring). It further indicates that the association between offspring size and number was shaped differently in the evolutionary history among vertebrates, for example, by differences in habitats, biotic interactions, lifestyle (e.g., terrestrial or aquatic), parity mode or parental care.

### 4.4 Conclusions

Our findings suggest that adult body mass correlates more strongly with amphibian life history traits than previously thought. Nevertheless, many traits characterising offspring development from egg to metamorphosis do not scale to body mass, which could indicate that these traits are strongly influenced by environmental conditions. The two biological time–related traits, age at maturity and maximum longevity, seem to be stronger determined by intrinsic processes, that is, by metabolic rate, as the MDH was supported for both traits when using a metabolic scaling exponent of 0.88. None of our allometric models on traits provided evidence for the MTE, questioning that a metabolic scaling exponent of ¾ (Kleiber's law) is appropriate to explain scaling of amphibian life history traits. Our fast‐slow continua inferred for all amphibians together and for each of the orders frogs and salamanders indicate that amphibians have diversified their life history strategies along two rather orthogonal axes. All first axes represent a fast‐slow continuum, along which body mass, age at maturity, maximum longevity, egg volume and clutch size correlate positively among each other. As such a first, fast‐slow axis was also found in fishes, reptiles, birds and mammals, it seems to be the most important axis of variability in vertebrate life history strategies. The second axes found for frogs, salamanders and across amphibians all comprise a trade‐off between egg volume and clutch size which is rather orthogonal to the fast‐slow continuum and thus nearly independent of adult body mass, age at maturity and maximum longevity. We suggest that this trade‐off is key for reproductive and ecological diversification in amphibians and could have enabled species to occupy a broad range of ecological niches without changing their adult body mass.

## Author Contributions

**Benjamin Cejp:** data curation (lead), formal analysis (lead), writing – original draft (equal), writing – review and editing (equal). **Eva Maria Griebeler:** conceptualization (lead), supervision (lead), writing – original draft (equal), writing – review and editing (equal).

## Conflicts of Interest

The authors declare no conflicts of interest.

## Data Availability

All data used are listed in the manuscript and its Appendix A.

## Appendix A

**TABLE A1:** The amphibian life history traits in our dataset. Shown are the trait names as used in the text and figures, their definitions and units.

Trait name	Definition	Unit
Egg diameter	Egg diameter	mm
Egg volume	Egg volume; calculated from egg diameter using the formula egg volume = 4/3 π r^3^, with r being egg diameter/2 in cm	cm^3^
Clutch size	Number of eggs in a clutch	
Clutch volume	Clutch volume; calculated from egg volume times clutch size	cm^3^
Annual clutch number	Annual clutch number; number of clutches that a female lays per year	
Annual clutch volume	Annual clutch volume; calculated from clutch volume times annual clutch number	cm^3^
Incubation time	Incubation time; time from egg deposition until hatching	days
TL of hatchlings	Total length of hatchlings	mm
SVL of hatchlings	Snout‐vent‐length of hatchlings	mm
Larval period	Duration of the larval period; time from hatching until metamorphosis	days
TL at metamorphosis	Total length at metamorphosis (Gosner stages 39–42)	mm
SVL at metamorphosis	Snout‐vent length at metamorphosis (Gosner stages 39–42)	mm
Juvenile mass	Mass of juveniles; measured directly after metamorphosis is completed	g
Age at maturity	Age at sexual maturity	years
Size at maturity	Size at sexual maturity	mm
Maximum longevity	Maximum longevity	years

**TABLE A2:** Results of phylogenetic PCAs conducted with all amphibians (*n* = 263), frogs (*n* = 166) and salamanders (*n* = 97).

Group	Amphibians		Frogs		Salamanders	
Axis	PC1	PC2	PC1	PC2	PC1	PC2
Adult body mass	0.78	−0.07	0.82	0.05	0.69	−0.22
Egg volume	0.47	0.74	0.31	0.85	0.69	0.51
Clutch size	0.43	−0.80	0.56	−0.66	0.18	−0.93
Age at sexual maturity	0.71	0.11	0.69	0.10	0.78	0.16
Maximum longevity	0.72	−0.05	0.67	−0.01	0.79	−0.20
Variance explained	41.0%	23.9%	40.3%	23.3%	44.3%	24.8%

*Note:* The phylogenetic signal assessed from Pagel's *λ* (Pagel 1999) is 0.81 for all amphibians, 0.75 for frogs and 0.78 for salamanders. Loadings of traits and variances explained by axes are shown for the first two axes based on the Kaiser–Guttman criterion (Guttman 1954).
